# E35 ablates acute leukemia stem and progenitor cells in vitro and in vivo

**DOI:** 10.1002/jcp.29457

**Published:** 2020-01-21

**Authors:** Yingyu Chen, Jing Zheng, Donghui Gan, Yanxin Chen, Na Zhang, Yuwen Chen, Zhenxing Lin, Wenfeng Wang, Haijun Chen, Donghong Lin, Jianda Hu

**Affiliations:** ^1^ Department of Hematology Fujian Institute of Hematology Fujian Medical University Union Hospital Fuzhou Fujian China; ^2^ Department of Clinical Laboratory School of Medical Technology and Engineering Fujian Medical University Fujian China; ^3^ Key Laboratory of Molecule Synthesis and Function Discovery (Fujian Province University), College of Chemistry Fuzhou University Fuzhou Fujian China; ^4^ Department of Hematology The Affiliated Hospital of Putian University Putian Fujian China

**Keywords:** acute leukemia, Akt/mTOR, apoptosis, E35, leukemia stem/progenitor cells, xenograft model

## Abstract

Leukemia stem cells (LSCs) have critical functions in acute leukemia (AL) pathogenesis, participating in its initiation and relapse. Thus, identifying new molecules to eradicate LSCs represents a high priority for AL management. This work identified E35, a novel Emodin derivative, which strongly inhibited growth and enhanced apoptosis of AL stem cell lines, and primary stem and progenitor cells from AL cases, while sparing normal hematopoietic cells. Furthermore, functional assays in cultured cells and animals suggested that E35 preferentially ablated primitive leukemia cell populations without impairing their normal counterparts. Moreover, molecular studies showed that E35 remarkably downregulated drug‐resistant gene and dramatically inhibited the Akt/mammalian target of rapamycin signaling pathway. Notably, the in vivo anti‐LSC activity of E35 was further confirmed in murine xenotransplantation models. Collectively, these findings indicate E35 constitutes a novel therapeutic candidate for AL, potentially targeting leukemia stem and progenitor cells.

## Graphical abstract

Leukemia stem cells have critical functions in acute leukemia (AL) pathogenesis, participating in its initiation and relapse. This work identified E35, a novel Emodin derivative, ablated primitive leukemia cell populations without impairing their normal counterparts, indicating E35 constitutes a novel therapeutic candidate for AL.

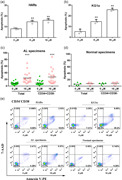

## 1 INTRODUCTION

Acute leukemia (AL) represents a clonal hematopoietic stem cell (HSC) dysregulation marked by arrested differentiation, unsuitable growth of immature progenitor cells. Persistence of leukemia stem cells (LSCs) constitutes an important factor that contributes to both initiation and relapse in patients with AL. Meanwhile, LSCs typically are quiescent and not susceptible to most conventional chemotherapy regimens. In addition, currently available regimens might not efficiently distinguish normal from malignant cells (Akbarzadeh et al., 2019; Bonnet & Dick, 1997; Guan, Gerhard, & Hogge, 2003; Guzman et al., 2005; Lapidot et al., 1994; van Rhenen et al., 2005). Thus, identifying novel molecules that could specifically target LSCs represents a high priority for leukemia therapy.

Emodin, a plant‐derived anthraquinone, has recently attracted increasing attention because of its overt pharmacological features in cancer therapy (Fu et al., 2012; Liu et al., 2016; Subramaniam et al., 2013). Brown, Bellon, and Nicot (2007) and our previous studies demonstrated the promising anti‐leukemic activities of Emodin in different types of hematological malignancies (Chen et al., 2014; Chen, Mei et al., 2018). We showed that Emodin can enhance all‐trans retinoic acid (ATRA)‐associated differentiation and induce apoptotic death in acute myeloid leukemia (AML) cells (Chen et al., 2014). In addition, our team found that Emodin may significantly increase the chemosensitivity of resistant AML cells to cytarabine (Ara‐C; Chen, Gan et al., 2018). Our recent study further demonstrated that TP53 and PI3K are the targets by which Emodin may function in diffuse large B cell lymphoma (Chen, Mei et al., 2018). However, Emodin is poorly soluble, which limits its potential application in clinic.

For identifying novel agents possessing enhanced drug‐like features, we recently designed, synthesized, and biologically evaluated a series of Emodin derivatives with quaternary ammonium salt (China Patent ZL201110045332.8, ZL201510406814.X, ZL201510868080.7)(Hu et al., 2019; Li et al., 2015; Shao et al., 2012; Wang et al., 2012). Among these compounds, E35 (C_34_H_50_ BrNO_5_·H_2_O, MW: 631.29), which was obtained by introducing a long quaternary ammonium salt containing a long carbon chain to Emodin, exhibited potent anti‐leukemia effects with low half maximal inhibitory concentration (IC50) values and markedly enhanced aqueous solubility. The positive charge of quaternary ammonium salt makes it easy for E35 to enter the mitochondria of cancer cells preferentially. Meanwhile, the long carbon chain renders E35 lipophilic and allows it to pass through the mitochondrial membrane easily (Hu et al., 2019; Li et al., 2015). E35 was interestingly found to have significant inhibitory effects on chronic myeloid leukemia (CML) 32Dp210‐T315I cells harboring T315I mutation in our previous report (Li et al., 2015). It is well‐accepted that the T315I gatekeeper mutation promotes resistance to both imatinib and second‐generation tyrosine kinase inhibitors, such as nilotinib and dasatinib in patients with CML (O'Hare et al., 2005; Tamai et al., 2018). According to its properties, E35 attracts increasing attention as a potential therapeutic for leukemia. Hence, we sought to further determine the biological significance of E35 in AL cells, especially in AL stem/progenitor cells. The present study demonstrated that LSCs are extremely sensitive to E35 treatment and undergo extensive apoptosis. Importantly, E35 efficiently suppressed human AL stem and progenitor cells, with no overt toxic effects on normal hematopoietic cells in culture or mice. These findings suggest E35 represents a potential new drug for treating hematologic cancers, particularly targeting AL stem/progenitor cells.

## 2 MATERIALS AND METHODS

### 2.1 Cell culture

Human multidrug resistant HL‐60/ADR cells and HL‐60 cells were from the Institute of Hematology, Chinese Academy of Medical Sciences. Highly malignant leukemia HL‐60/H3 cells were obtained from HL‐60 xenografts in the nude mouse model. ATRA‐resistant MR2 and NB4 cells were provided by Shanghai Institute of Hematology, Shanghai Ruijing Hospital, China. U937, CEM, Jurkat, Molt‐4, and CA46 cells were provided by CCTCC (China Center for Type Culture Collection, China). Cell culture followed the procedures described in our previous study (Chen et al., 2014). Human leukemic stem‐like KG1a cells (>95% CD34^+^ CD38^−^, American Type Culture Collection, ATCC) were maintained in Iscove's Modified Dulbecco's medium containing 10% fetal bovine serum (Gibco‐BRL) at 37℃ in a humid incubator containing 5% CO_2_. CD34^+^ cells enriched from HL‐60/ADR cells were termed HARs. E35 stock solution was prepared as reported in our previous study (Li et al., 2015).

### 2.2 Primary cell isolation

Peripheral blood was collected from 33 primary patients with AL. The specimens contained ≥70% blasts before any manipulation. All patients had AL, as described by standard French‐American‐British and WHO criteria. Cases and healthy volunteers provided informed consent, and the study had approval from the institutional review board of Fujian Medical University Union Hospital, and was carried out according to the Declaration of Helsinki. Normal control samples were from six healthy HSC donors upon granulocyte colony‐stimulating factor mobilization. Peripheral blood mononuclear cells (PBMCs) were purified by Ficoll‐Paque density‐gradient separation. The percentages of CD34^+^ cells in samples ranged from 4.0% to 97.6%. CD34^+^ cells were further selected from PBMCs with CD34 MicroBead Kit (MiltenyiBiotec). CD34^+^ cell populations with a purity >90% were used in all experiments. Primary cells were incubated with medium without serum supplementation for 1 hr before E35 treatment (Lansdorp & Dragowska, 1992).

### 2.3 Drug susceptibility assessment

Drug susceptibility was assessed by the 3‐(4,5‐dimethylthiazol‐2‐yl)‐2,5‐diphenyltetrazolium bromide (MTT) assay as previously reported by our team (Chen et al., 2014; Chen, Gan et al., 2018). Cells seeded in 96‐well plates (Costar) for 0‐72 hr were incubated with or without E35 at 37℃ in a humidified incubator containing 5% CO_2_. MTT (5 mg/ml, Sigma) in phosphate‐buffered saline was supplemented (10 µl/well), followed by 4 hr of incubation. After supernatant removal, dimethyl sulfoxide was employed to dissolve formazan crystals. All experiments were carried out in triplicate. Absorbance was assessed on a spectrophotometer (STAT FAX‐2100) at 492/630 nm. Cell survival and inhibitory rates were derived as (optical density [OD]_treated_/OD_control_) × 100% and (1−OD_treated_/OD_control_) × 100%, respectively. IC50 was determined by the Logit method.

### 2.4 Methylcellulose colony‐forming assay

A total of 500 CD34^+^ cells were seeded in 6‐well plates with 1 ml per well of complete methylcellulose medium supplemented with recombinant cytokines (MethoCult H4435; Stem Cell Technologies), and treated or not with E35. The colonies (>40 cells) were counted after 14 days of culture. The average percentages of colony‐forming units (CFUs) were obtained based on nontreated cells.

### 2.5 Nonobese diabetic/severe combined immunodeficiency mouse repopulating cell assay

Nonobese diabetic/severe combined immunodeficiency (NOD/SCID) mice (6–8 weeks; Shanghai Slac Laboratory Animal Co., Ltd.) were intravenously administered 25 mg·kg^−1^·day^−1^ busulfan (DSM Pharmaceuticals, Inc.) on days −2 and −1 before transplantation. Primary AL cells and healthy donor cells were incubated with 16 µM E35 or not for 18 hr, and then transplanted into busulfan preconditioned NOD/SCID mice (1 × 10^7^ cells/mouse). After 8 weeks, bone marrow cells were collected and incubated with antihuman CD45 antibodies (BD Biosciences). Human cells in various recipients were detected flow‐cytometrically.

### 2.6 Flow cytometric assessment of apoptosis and cell cycle distribution

HARs and KG1a cells were harvested after exposure to E35 for 24 hr, and underwent staining with CD34‐APC and CD38‐FITC antibodies detecting surface molecules (Becton Dickinson). Apoptotic rates were assessed using an Annexin V‐phycoerythrin (PE)/7‐amino‐actinomycin (7‐AAD) staining kit (Becton Dickinson) as directed by the manufacturer. Flow cytometry was then employed for analysis on a BD Verse (Becton Dickinson). To analyze the cell cycle distribution, cells underwent treatment with E35 for 48 hr. Then, single cell suspensions underwent fixation with 70% chilled ethyl alcohol for 2 hr and incubated with RNase A at 37℃ for 30 min, and incubated with propidium iodide (KeyGEN BioTECH) at 4℃ for 30 min. Flow cytometric assessment was carried out to obtain cell amounts in various phages (G0/G1, S, and G2/M), respectively.

### 2.7 Quantitative real‐time polymerase chain reaction

HARs and KG1a cells were administered E35 for 24 hr. Total RNA was obtained with RNeasy Mini Kit (Qiagen) according to the kit's specifications. RNA amounts and purity were assessed by UV spectrophotometry (Nanodrop). Reverse transcription was performed with OligodT primers and reverse transcriptase (Promega). Quantitative real‐time polymerase chain reaction (qRT‐PCR) was performed with SYBR Green on an ABI prism 7700 sequence detection system (Applied Biosystems) using Real Master Mix kit (Tiangen, China). Table 1 lists primer sequences for GAPDH, MDR1, MRP1, TopⅡβ, GSTπ, and BCL‐2 detection by qRT‐PCR. Relative messenger RNA (mRNA) amounts were determined by the $2^{-\Delta \Delta C_{t}}$ method, and normalized to GAPDH expression (Lin et al., 2013; Wang et al., 2015).

**Table 1:** The primer sequence for qRT‐PCR

Gene	Sequence
GAPDH	F: 5′‐CCACCATGGAGAAGGCTGGGGCTCA‐3′
	R: 5′‐ATCACGCCACAGTTTCCCGGAGGGG‐3′
MDR1	F: 5′‐CCCATCATTGCAATAGCAGG‐3′
	R: 5′‐GTTCAAACTTCTGCTCCTGA‐3′
MRP1	F: 5′‐ATCGTCATGAGTGGCGGCAA‐3′
	F: 5′‐ACTGTCCGTCACCAGCATGC‐3′
TopⅡβ	F: 5′‐GCTGTGGATGACAACCTCC‐3′
	R: 5′‐CTGTGTTTCTGTCCACTAC‐3′
GSTπ	F: 5′‐CGGGGCGGGACCACCCTTAT‐3′
	R: 5′‐CACGGTGTAGGGCGGCATGG‐3′
BCL‐2	F: 5′‐ACGACTTCTCCCGCCGCTAC‐3′
	R: 5′‐CTGAAGAGCTCCTCCACCAC‐3′

Abbreviation: qRT‐PCR, quantitative real‐time polymerase chain reaction.

### 2.8 Western blot analysis

HARs and KG1a cells were administered increasing E35 amounts for 24 hr. Immunoblot was carried out upon cell treatment following reported protocols (J. Hu et al., 2011). Antihuman Procaspase‐9, Procaspase‐3, p‐Akt (Thr308), Akt, p‐4E‐BP1 (Thr70), 4E‐BP1 (53H11), p‐p70S6K (Thr389), and p70S6K primary antibodies were provided by Cell Signaling Technology; anti‐MRP1 primary antibodies from Boster Biological Technology (China), and anti‐MDR1, GSTπ, TopⅡβ (C‐12), BCL‐2, and β‐actin primary antibodies provided by Santa Cruz Biotechnology were also employed.

### 2.9 In vivo anti‐leukemic effects in AML xenograft model

KG1a cells were transduced with a lentiviral vector encoding the red fluorescent protein (RFP) gene. The efficiency of the stable transduction was monitored based on observations made by fluorescence microscopy. Around 2 × 10^6^ KG1a cells with stable‐RFP expression, namely KG1a‐R, were transplanted to 50 mg/kg busulfan preconditioned BALB/c‐nude mice. Five days after transplantation, the KG1a‐R xenograft models received 20 mg/kg E35 by intraperitoneal injection once a day for 14 days (*n* = 8). Saline injection was administered as a parallel control group (*n* = 8). KG1a‐R xenograft models administered E35 were monitored on an IVIS LUMINA II Imaging System (Caliper Life Sciences) at 10 weeks upon initial treatment. After euthanasia, bone marrow smears were obtained. Cell morphology was assessed microscopically upon Wright‐Giemsa staining. Bone marrow cell blocking (anti‐Fc receptor antibodies) was performed before incubation with CD34‐APC and CD38‐FITC monoclonal antibodies (Becton Dickinson). The animals were maintained in SPF conditions in the vivarium of Fujian Medical University. Animal studies had approval from the Institutional Animal Care and Use Committee of Fujian Medical University.

### 2.10 Statistical analysis

Data are mean ± standard deviation, and were evaluated by *t* test. GraphPad Prism 6.0 was employed for statistical analysis. Significance level was set at *p* < .05.

## 3 RESULTS

### 3.1 Various leukemia/lymphoma cell lines are more sensitive to E35 compared with Emodin

The effects of E35 on the growth and viability of 10 different AL cell lines were assessed, including six AML cell lines (HL‐60, HL‐60/ADR, HL‐60/H3, NB4, MR2, and U937 cells), three acute lymphoblastic leukemia cell lines (Molt‐4, CEM, and Jurkat cells) and the Burkitt lymphoma CA46 cell line. Cells were incubated for 72 hr with various E35 amounts. All cells were sensitive to E35 treatment, and cell proliferation was markedly inhibited in an E35 dose‐dependent way. The IC50 values of E35 in the 10 cell lines were between 1.48 ± 0.31 µM and 3.67 ± 0.69 µM, that is, 6.07‐ and 25.47‐fold lower than those of Emodin as previously reported by our team (Chen et al., 2014; Chen, Gan et al., 2018; Table 2).

**Table 2:** The comparison of different cell lines in response to Emodin and E35 treatment

Cell line	IC50, µM	
	Emodin^a^	E35
HL‐60	23.18 ± 0.87	2.17 ± 0.05
HL‐60/ADR	24.09 ± 1.72	1.53 ± 0.04
HL‐60/H3	30.56 ± 4.43	1.87 ± 0.15
NB4	37.99 ± 2.30	2.67 ± 0.58
MR2	34.01 ± 2.40	2.11 ± 0.19
U937	29.96 ± 1.66	3.38 ± 0.57
CA46	37.70 ± 1.16	1.48 ± 0.31
Molt‐4	22.28 ± 3.07	3.67 ± 0.69
CEM	28.41 ± 3.13	2.36 ± 0.86
Jurkat	39.17 ± 1.69	2.22 ± 1.63

*Note*: The results were presented as mean ± *SD* of three independent experiments.

Abbreviations: IC50, half maximal inhibitory concentration; *SD*, standard deviation.

^a^ (Chen et al., 2014; Chen et al., 2018).

### 3.2 E35 preferentially targets primitive leukemia cells and spares non‐diseased hematopoietic cells

**Figure 1:**
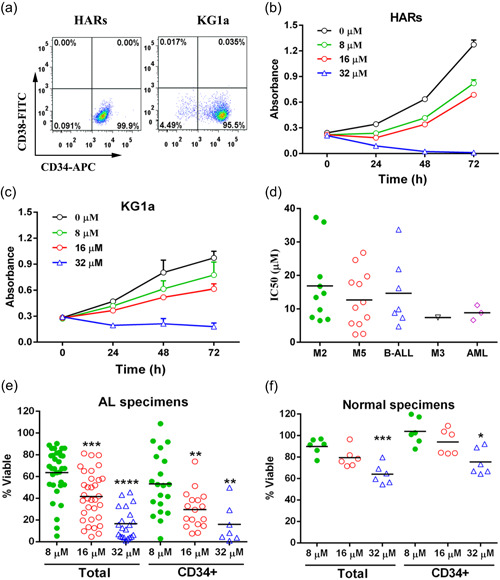
Cell viability of AL and normal samples after E35 administration, as measured by the MTT assay. (a) HARs cells and KG1a cells bearing more than 95% CD34^+^CD38^−^ immunophenotype. (b and c) Proliferation of HARs and KG1a cells incubated with or without E35 at the indicated times and levels. All assays were performed in triplicate. (d) Primary AL cells were administered increasing E35 amounts (5–80 µM) for 72 hr. IC50 values in 33 AL patient specimens with distinct FAB classification subtypes. (e and f) Cell viability rates of primary human AL cells administered the indicated concentrations of E35 for 72 hr. Total AL versus CD34^+^ cells were assessed. Viability was relative to untreated control samples. Each symbol denotes one sample. Triplicate values were obtained and averaged per sample. *p < .05, **p < .01, ***p < .001, ****p < .0001 versus 8 µM E35 group. FAB, French‐American‐British; IC50, half maximal inhibitory concentration; MTT, 3‐(4,5‐dimethylthiazol‐2‐yl)‐2,5‐diphenyltetrazolium bromide

HARs and KG1a cells (both bearing more than 95% CD34^+^CD38^−^ immunophenotype, Figure 1a were administered E35 at different levels. Cell viability was determined by the MTT assay after different exposure times. As shown in Figure 1b,c, E35 dose‐dependently reduced viability in both HARs and KG1a cells. To assess whether comparable effects are valid for primary AL cells, 33 specimens from AL patients with different subtypes were administered various doses of E35 (5–80 µM) for 72 hr. All of the primary cells from AL patients with different subtypes were affected by E35, with IC50 values of 13.86 ± 9.35 µM (Figure 1d). Figure 1e shows that primary AL cells were dose‐dependently inhibited by E35. At E35 concentrations of 8, 16, and 32 µM, mean viability rates in total cell populations were 63.60 ± 3.87% (*n* = 33), 41.68 ± 3.90% (*n* = 33) and 16.63 ± 3.09% (*n* = 21), respectively. Next, cell death in CD34^+^ primitive AL cells was assessed. Cells administered E35 at 8, 16, and 32 µM showed 53.12 ± 6.32% (*n* = 20), 29.68 ± 4.18% (*n* = 17), and 15.98 ± 6.71% (*n* = 7) mean viability rates, respectively. To verify E35 specificity for cancer cells, normal hematopoietic cells were examined. As shown in Figure 1f, both total and CD34^+^ cells from normal samples showed no overt viability reduction upon treatment with 8 and 16 µM E35 (89.86 ± 3.05% vs. 79.43 ± 3.73%, respectively, *p* = 0.06, *n* = 6 for total cells; and 103.8 ± 5.13% vs. 94.09 ± 4.79%, respectively, *p* = 0.195, *n* = 6 for CD34^+^ cells). When administered E35 as high as 32 µM, the results showed that viability in total and CD34^+^ healthy cells was 64.04 ± 3.81% (*n* = 6) and 75.33 ± 4.96% (*n* = 6), respectively, that is, 3.9‐fold and 4.7‐fold higher than those observed in total and in CD34^+^ AL cells administered 32 µM E35.

### 3.3 E35 decreases leukemic but not normal stem and progenitor cell activity

**Figure 2:**
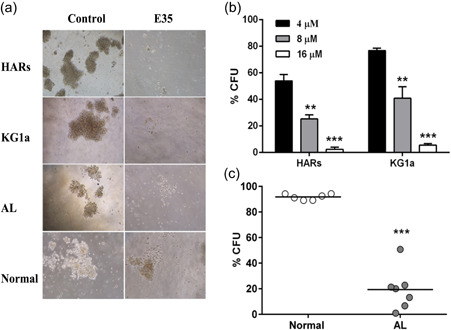
In vitro colony formation abilities of AL and normal cells administered E35. (a and b) HARs and KG1a cells were administered increasing amounts (4–16 µM) of E35 for 14 days. Average percentages of colony‐forming units (CFUs) were based on untreated controls. Data are mean ± *SD* of three experiments performed independently. **p < .01, ***p < .001 versus 4 µM E35 group. (a and c) CD34^+^ cells retrieved from primary human AL cells and normal cells were administered 16 µM E35 for 14 days. Average percentages of CFUs were based on untreated controls. ***p < .001 versus normal group. All assays were performed in triplicate. Representative CFU images acquired under a microscope are shown in panel (A). AL, acute leukemia; *SD*, standard deviation

The in vitro colony formation assay was employed to assess whether E35 altered colony formation in primitive cells. Figure 2a,b show that CFUs were dramatically reduced in both HARs and KG1a cells exposed to increasing doses (8 and 16 µM) of E35. For comparison, we further treated CD34^+^ cells from healthy donors (*n* = 6) and AL cases (*n* = 7) with 16 µM E35 for 14 days and assessed CFUs. The results revealed that CFUs from normal donors were slightly decreased by about 20%; however, CFUs from patients with AL were strongly inhibited by more than 90% upon E35 treatment (Figure 2a,c).

### 3.4 E35 reduces NOD/SCID repopulating ability in AL cells but not normal cells

**Figure 3:**
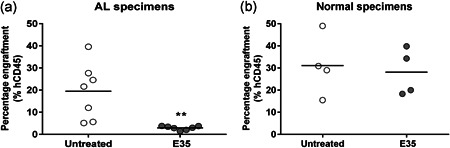
Hematopoietic stem/progenitor cell functional assays for E35 treated cells in vivo. Rates of engraftment in NOD/SCID mice transplanted human AL (a) or normal (b) cells following 18 hr of incubation with/without 16 µM E35. Human cell engraftment levels were analyzed by flow cytometry. Each symbol denotes an individual bone marrow specimen at 8 weeks following transplantation. **p < .01 versus untreated group. AL, acute leukemia; NOD/SCID, nonobese diabetic/severe combined immunodeficiency

NOD/SCID mouse xenograft assays were performed to assess whether E35 targets functionally defined leukemia progenitor/stem cells. Primary AL and normal cells after an 18‐hr treatment with E35 were administered to immunodeficient NOD/SCID mice. Bone marrow cells were collected and examined for human‐derived CD45 cells in recipients at 8 weeks after transplantation. Figure 3a shows that E35 treatment markedly reduced human leukemic cell engraftment in NOD/SCID mice. The engraftment levels of seven independent AL specimens in the recipients were decreased by 85% compared with the untreated group (*p* = .005, *n* = 7). In contrast, hCD45 cell assessment in animals treated versus untreated healthy cells showed no statistically significant difference (Figure 3b; *p* = .742, *n* = 4), indicating that E35 specifically targets AL stem/progenitor cells with no effect on the engraftment potential of normal primitive cells.

### 3.5 E35 specifically induces apoptotic response in primitive AL cells

**Figure 4:**
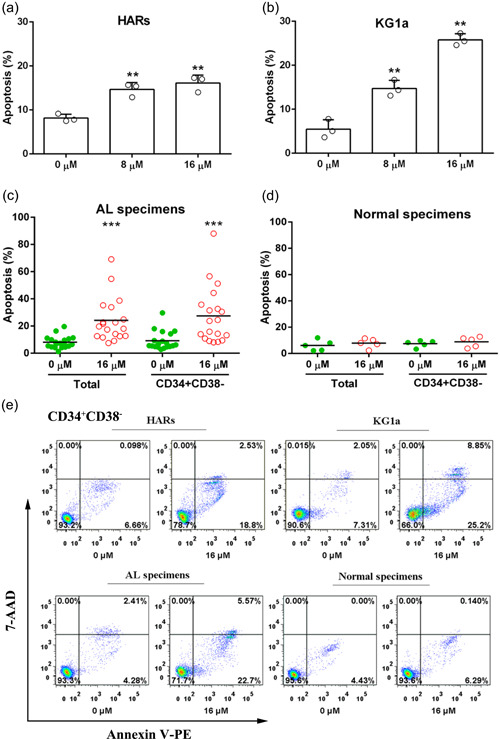
E35 induces apoptosis in AL stem and progenitor cells but not normal cells. Early apoptosis (Annexin V^+^ 7‐AAD^−^) was detected flow‐cytometrically following Annexin V‐PE/7‐AAD staining. (a, b) HARs and KG1a cells were administered 8 and 16 µM E35 for 24 hr, respectively. Data are mean ± *SD* of three experiments performed independently. **p < .01 versus vehicle control. (c, d) Primary cells from 19 patients with AL and normal cells from five healthy stem cell donors were incubated with or without 16 µM E35 for 24 hr, respectively. The rates of early apoptotic cells among total and CD34^+^CD38^−^ cells were assessed flow‐cytometrically. Each symbol denotes one specimen. ***p < .001 versus vehicle control. (e) Representative flow‐cytograms of CD34^+^ CD38^−^ cells. Dot plots showing 7‐AAD versus Annexin V‐PE. 7‐AAD, 7‐amino‐actinomycin; AL, acute leukemia; PE, phycoerythrin; *SD*, standard deviation

To explore the underlying mechanism by which E35 affects AL stem and progenitor cells, we investigated whether treatment with E35 was associated with apoptotic induction in leukemic cells. As shown in Figure 4a,b, E35 dose‐dependently exerted apoptotic effects in both HARs and KG1a cells. The rates of early apoptosis (Annexin V‐PE^+^ 7‐AAD^−^) were 16.10 ± 1.05% and 25.80 ± 0.79%, respectively, when E35 was administered at 16 µM in HARs and KG1a cells for 24 hr. Similarly, robust apoptosis induction by E35 occurred in primary AL cells and LSCs. Early apoptotic cells were significantly raised to 24.18 ± 3.69% (*n* = 19) and 27.49 ± 4.80% (*n* = 19) in total and CD34^+^ CD38^−^ cell populations, respectively, after 24‐hr incubation with 16 µM E35 (*p* = .0002 and *p* = .0009 vs. untreated controls, respectively; Figures 4c,e). We also compared normal cells following the same treatment. The results showed that both total and CD34^+^CD38^−^ cells from healthy donors were basically unaffected by E35 compared with the control group (*p* = .494 and *p* = .513 vs. untreated control, *n* = 4; Figure 4d,e). Taken together, these findings suggested E35 promotes apoptosis specifically in primitive AL cells, but shows no toxicity to normal cells.

### 3.6 E35 increases the cell proportion in the G0/G1 phase and reduces G2/M phase cell amounts in HARs and KG1a cells

**Figure 5:**
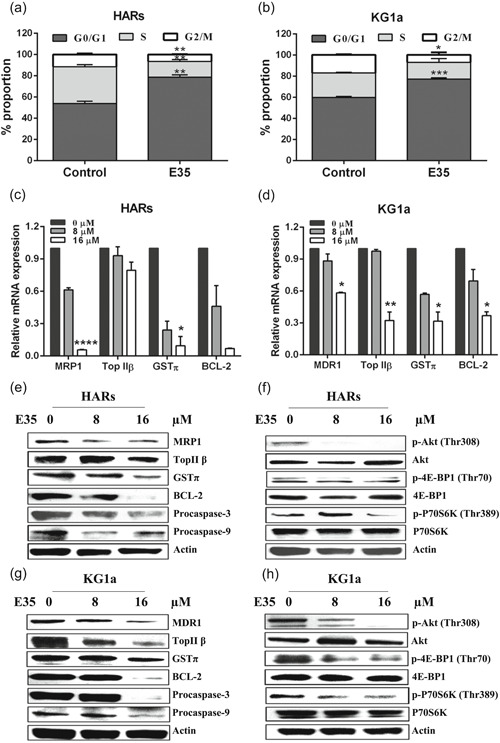
Effects of E35 on cell cycle distribution, mRNA, and protein expression levels. (a, b) HARs and KG1a cells were administered 16 µM E35 for 48 hr. Cell rates in various phases were assessed after incubation with propidium iodide (PI), respectively. Data are mean ± *SD* of three experiments performed independently. *p < .05, **p < .01, ***p < .001 versus vehicle control. (c, d) Total RNA was harvested from HARs and KG1a cells at 24 hr upon administration of increasing concentrations of E35. Quantitative real‐time PCR analysis showed E35 dose‐dependent decreases in mRNA levels of MRP1, MDR1, TopⅡβ, GSTπ, and BCL‐2. The mRNA expression levels are relative to control levels, considered to be 100% (1.0). All assays were repeated three times. *p < .05, **p < .01, ****p < .0001 versus 8 µM E35 group. (e–h) Total protein was extracted from HARs and KG1a cells at 24 hr after incubation with the indicated concentrations of E35. Western blot analysis demonstrated E35 dose‐dependent reductions of MRP1, MDR1, TopⅡβ, GSTπ, BCL‐2, Procaspase‐3 and Procaspase‐9 protein, and Akt, 4E‐BP1 and P70S6K phosphorylation levels. β‐Actin was employed as an internal reference. mRNA, messenger RNA; PCR, polymerase chain reaction; *SD*, standard deviation

E35 induced G0/G1 phase arrest in HARs and KG1a cells after 48‐hr incubation at a dose of 16 µM. Compared with control cells, treatment with E35 significantly increased the cell amounts in the G0/G1 phase (78.79 ± 2.18% vs. 53.92% ± 2.27%, *p* = .0014 for HARs; and 77.24 ± 1.35% vs. 59.91% ± 1.03%, *p* = .0005 for KG1a cells). The S phase cell rates were similar in both groups of KG1a cells (15.81 ± 3.60% vs. 23.09 ± 0.66%; *p* = .117), while there was a dramatic reduction of cell proportion in the S phase in HARs after treatment with E35 (14.64 ± 1.84% vs. 34.68 ± 1.86%; *p* = .0016). The proportion of G2/M phase cells upon E35 administration was markedly reduced in comparison with the control value (6.58 ± 0.40% vs. 11.40% ± 0.91%, *p* = .0083 for HARs; and 7.21 ± 2.37% vs. 17.25 ± 0.60%, *p* = .015 for KG1a cells; Figure 5a,b).

### 3.7 E35 downregulates drug resistance genes and inhibits the Akt/mammalian target of rapamycin signaling pathway

To evaluate the molecular consequences of E35 treatment in primitive leukemia cells, we examined the expression changes of drug‐resistant genes by qRT‐PCR and immunoblot, respectively. As depicted in Figure 5c–e and Figure 5g, E35 dose‐dependently decreased the mRNA and protein levels of MDR1, MRP1, TopⅡβ, GSTπ, and BCL‐2 in HARs and KG1a cells after 24‐hr of incubation. Meanwhile, the expression levels of Procaspae‐9 and Procaspae‐3 were remarkably lower in the 16 µM E35 treatment group than those of untreated control cells. Next, we examined whether E35 affected Akt/mammalian target of rapamycin (mTOR) signaling. Akt (Thr308), p70S6K (Thr389), and 4E‐BP1 (Thr70) phosphorylation levels were then evaluated in HARs and KG1a cells after E35 treatment. Figures 5f,h show that E35 markedly and dose‐dependently blunted p‐Akt, p‐p70S6K, and p‐4E‐BP1 amounts in HARs and KG1a cells, while total Akt, p70S6K, and 4E‐BP1 amounts were almost unaffected. Thus, inhibition of the Akt/mTOR axis is associated with the anti‐leukemic activity of E35.

### 3.8 KG1a cell response to E35 treatment in the xenograft mouse model

**Figure 6:**
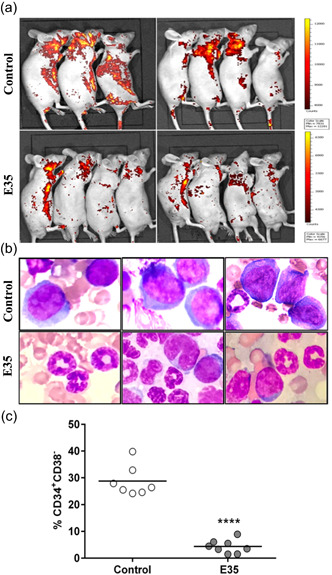
In vivo therapeutic effects of E35 in KG1a‐R xenograft mice. Nude mice harboring KG1a‐R xenografts were randomized into two groups and intraperitoneally administered 20 mg/kg E35 or vehicle once daily for 2 weeks. All animals were followed up for 10 weeks after the initial treatment with E35. (a)The leukemic burden was assessed on an IVIS LUMINA II Imaging System. (b) Harvested bone marrow (BM) cells were stained by the Wright‐Giemsa method. (c) The percentages of CD34^+^CD38^−^ KG1a‐R cells in BM were measured by flow cytometry. ****p < .0001 versus vehicle control

The in vivo anti‐leukemic effect of E35 was further investigated based on leukemic stem cell‐like KG1a‐R xenograft models. Animals were imaged on an IVIS LUMINA II Imaging System at the 10th week after treatment initiation. KG1a‐R xenograft mice presented a strong therapeutic response to E35. Bioluminescent imaging results revealed a dramatic reduction of tumor burden in recipients that received E35 treatment (Figure 6a). Wright‐Giemsa‐stained sections showed elevated immature blast cell infiltration in the bone marrow from saline control mice. In contrast, bone marrow samples from E35‐conditioned mice were dominated by more mature myeloid cells at various differentiation levels (Figure 6b). Flow cytometric analysis was also performed to track human KG1a‐R cells in the bone marrow from individual mice. As shown in Figure 6c, the percentages of CD34^+^CD38^−^ KG1a‐R cells in recipients were markedly reduced following E35 treatment in comparison with control values (*p* < .0001). Of note, all treated animals appeared healthy; none of them appeared to succumb to therapeutic toxicity, and all survived to the end of observation. In contrast, one mouse in the saline control group died due to rapid disease progression (data not shown). Hence, the in vivo study further confirmed the potential of E35 for the eradication of leukemic stem/progenitor cells.

## 4 DISCUSSION

AL arises from immature cells in the bone marrow. It represents a severe and common hematologic malignancy. Because LSCs play a critical role in AL initiation, multidrug resistance and recurrence of leukemia (Bonnet & Dick, 1997; Lapidot et al., 1994; Ratajczak, Bujko, Mack, Kucia, & Ratajczak, 2018; van Rhenen et al., 2007), selectively targeting LSCs has been identified as a promising strategy for leukemia treatment (Baquero et al., 2018; Ding et al., 2016; Guzman et al., 2014; Liu et al., 2013; Tremblay et al., 2018). Here, we provide the first in vitro and in vivo evidence that E35, a novel Emodin derivative, preferentially eradicates AL stem and progenitor cells.

We recently demonstrated that Emodin has several properties, including inhibiting leukemic cell proliferation, sensitizing resistant leukemic cells to chemotherapeutic agents and suppressing diffuse large B cell lymphoma. In this study, we increased the antileukemic properties of Emodin by designing a more pharmacologically potent derivative, E35, which retained the key features of the parent molecule. The IC50 values of E35 in 10 different leukemia/lymphoma cell lines were between 6.07‐ and 25.47‐fold lower than those of Emodin. We then examined whether E35 may eliminate AL stem and progenitor cells. According to previous reports, KG1a cells with the similar LSC phenotype of CD34^+^CD38^−^ are considered an ideal cell model for studying LSCs (Ding et al., 2016; Liu et al., 2013; Weng, Zeng, Huang, Fan, & Guo, 2015). We thus selected KG1a cells and HARs (CD34^+^ cells enriched from multidrug‐resistant HL‐60/ADR cells) to perform in vitro experiments. Our results showed that both cell proliferation and colony formation were dramatically inhibited after E35 treatment, indicating that E35 may effectively ablate LSCs.

To generate data more relevant to clinical application, primary AL cells were assessed. Peripheral blood specimens from 33 AL patients with different subtypes and seven healthy donors were collected in this study. Interestingly, we found that E35 was able to decrease cell viability in primary AL stem and progenitor cells as well as the total blast cells. Single E35 exposure was sufficient to induce a strong apoptotic response in both LSC cell lines and primary LSCs. Functional assays demonstrated that E35 had the ability to target AL progenitor and stem cell populations as assessed by in vitro colony formation assay and in vivo engraftment assays. More important, normal human HSCs and progenitor cells were almost unaffected by the tested concentrations of E35, indicating that E35 has the tremendous potential as an antileukemic substance, specifically targeting primitive AL stem/progenitor cells.

We further explored the underlying molecular mechanisms evoked by E35 treatment in KG1a cells. It is known that nuclear factor κB (NF‐κB) modulates mesenchymal stem cell accumulation at tumors. Inhibition of NF‐κB activity might contribute to apoptotic induction in LSCs (Guzman et al., 2001; Guzman et al., 2002; Guzman et al., 2014; Ji et al., 2016; Jin et al., 2017; Uchibori et al., 2013). Mohammadi et al. (2016) reported that the Akt/mTOR/PTEN/β‐catenin/NF‐kB cascade plays important roles in controlling osteopontin‐mediated LSC survival and enrichment. A recent study by Zeng et al. (2016) revealed that MLN0128, a second‐generation mTOR kinase inhibitor currently in clinical development, might promote AML stem/progenitor cell killing through selective blockade of AKT/mTOR signaling. In the current study, we demonstrated that E35 dose‐dependently abrogated Akt phosphorylation in KG1a cells. Meanwhile, it inhibited the activation of 4E‐BP1 and p70S6K, two important downstream targets of the mTOR signaling pathway, in the same manner. Our findings indicated that exposure of LSCs to E35 may result in blocked AKT/mTOR pathway, which was consistent with Zeng's at al findings. Previous studies have also shown that KG1a cells exhibit multiple drug‐resistance mediated by ATP‐binding cassette transporters (Chen, Lee, Kang, Minden, & Zhang, 2018; Ding et al., 2016; Fuchs, Daniel, Sadeghi, Opelz, & Naujokat, 2010; Rao et al., 2011). In this study, we found that HARs and KG1a cells in response to E35 showed dramatic reductions in both mRNA and protein expression levels of MDR1, MRP1, GSTπ, TopⅡβ, and BCL‐2. These molecules are associated with drug‐resistance in AL (Lin et al., 2013; Wang et al., 2015). Therefore, we speculate that blockade of the AKT/mTOR pathway and downregulation of drug‐resistant genes contributes to the efficacy of E35 for LSC inhibition.

Moreover, upon E35 administration, more HARs and KG1a cells underwent G0/G1 phase arrest. The cell cycle comprises the G1 (growth/chromosomal preparation for replication), S (DNA replication), G2 (preparation for mitosis), and M (mitosis) phases. Preventing the S phase malignant cells from progressing to the M phase would help control cancer growth. The current work showed that E35 at 16 µM exclusively arrested HARs and KG1a cells in the G0/G1 phase in comparison with vehicle controls. The rates of HARs and KG1a cells in the G0/G1 phase were remarkably elevated in the E35 exposure group, which indicates that E35 prevents G1 phase cells from progressing to the M phase, interfering with cell proliferation. The results of the cell cycle assay are concordant with those of E35‐medicated growth inhibition and apoptotic induction in vitro, which also corroborate findings in several studies assessing novel therapeutic strategies for leukemia and LSCs (Chen et al., 2016; Park et al., 2018; Rahimian, Mahdavi, Rahbarghazi, & Charoudeh, 2019; Schinke et al., 2015). However, these findings are preliminary, and it is worthwhile further investigating whether E35 may affect key cell cycle modulators, including Cyclin D1, p27, and CDK4/6.

Furthermore, we evaluated the in vivo anti‐leukemic activity of E35 using a human LSC‐like KG1a‐R xenotransplantation mouse model. All animal recipients survived at the 10th week during follow‐up after initial treatment with E35. RFP signals were markedly decreased in animals administered E35. Consistently, dramatically decreased rates of human‐derived CD34^+^CD38^−^ cells in the bone marrow were observed as measured by flow cytometry, concomitant to increased amounts of differentiated myeloid cells in bone marrow smears. Taken together, we provide the preliminary evidence that E35‐mediated anti‐LSC activity can occur in the xenograft model. It has been reported that different events jointly inhibit LSCs in vivo. For example, Ding et al. (2016) showed that alantolactone dose‐dependently induces apoptosis in KG1a cells via suppression of NF‐κB and its downstream target proteins, while its prodrug DMA‐alantolactone could greatly inhibit KG1a xenograft in vivo. Guzman et al. (2007) found that the anti‐LSC activity mediated by the dimethyl‐amino analog of parthenolide in canine models is strongly associated with induced oxidative stress responses and NF‐κB suppression. The latter authors also reported that Histone Deacetylase Inhibitor AR‐42 reduces Hsp90's ability to stabilize its oncogenic effectors, causing enhanced and specific cytotoxicity in LSCs (Guzman et al., 2014). Our findings demonstrated that the therapeutic response to E35 is mediated, at least in part, by differentiation induction in LSCs. However, the underpinning molecular mechanisms still need to be explored under in vitro and in vivo settings.

In summary, we identified E35 as a novel agent that can ablate AL cells at the bulk, stem, and progenitor cell levels. Going forward, further investigation in large animal models may enable greater success in applying this compound to clinical cases.

## CONFLICT OF INTERESTS

The authors declare that they have no conflict of interests.

## AUTHOR CONTRIBUTIONS

Y. C. initiated the research, performed the experiments, analyzed the data and wrote the manuscript. J. Z. and D. G. performed the experiments. Y. C., N. Z., Y. C., and Z. L. collected patient samples and analyzed some data. W. W. and H. C. assisted with the design and synthesis of E35. D. L. and J. H. provided administrative support. All authors approved the final manuscript.

## Data Availability

The data used to support the findings of this study are available from the corresponding author upon request.
